# *Mycoplasma agalactiae*, an Etiological Agent of Contagious Agalactia in Small Ruminants: A Review

**DOI:** 10.1155/2014/286752

**Published:** 2014-07-03

**Authors:** Amit Kumar, Anu Rahal, Sandip Chakraborty, Amit Kumar Verma, Kuldeep Dhama

**Affiliations:** ^1^Department of Veterinary Microbiology, Uttar Pradesh Pandit Deen Dayal Upadhayay Pashu Chikitsa Vigyan Vishwavidhyalaya Evum Go-Anusandhan Sansthan (DUVASU), Mathura 281001, India; ^2^Division of Pharmacology and Toxicology, Indian Veterinary Research Institute, Izatnagar 243122, India; ^3^Animal Resources Development Department, Pt. Nehru Complex, Agartala 799006, India; ^4^Department of Veterinary Epidemiology and Preventive Medicine, Uttar Pradesh Pandit Deen Dayal Upadhayay Pashu Chikitsa Vigyan Vishwavidhyalaya Evum Go-Anusandhan Sansthan (DUVASU), Mathura 281001, India; ^5^Division of Pathology, Indian Veterinary Research Institute, Izatnagar 243122, India

## Abstract

*Mycoplasma agalactiae* is one of the causal agents of classical contagious agalactia (CA), a serious, economically important but neglected enzootic disease of small ruminants. It occurs in many parts of the world and most notably in the Mediterranean Basin. Following the infection common complications are septicaemia, mastitis, arthritis, pleurisy, pneumonia, and keratoconjunctivitis. Primary or tentative diagnosis of the organism is based upon clinical signs. Various serological tests, namely, growth precipitation, immunofluorescence, complement fixation test, haemagglutination inhibition, agglutination, immunodiffusion, enzyme immunoassays, immunoelectrophoresis, blotting techniques, and others, are available. Molecular tools seem to be much more sensitive, specific, and faster and help to differentiate various strains. The real-time PCR, multiplex PCR, quantitative PCR, PCR-RFLP, MLST, and gene probes, complementary to segments of chromosomal DNA or 16S ribosomal RNA (rRNA), have strengthened the diagnosis of *M. agalactiae*. Both live attenuated and adjuvant (alum precipitated or saponified) inactivated vaccines are available with greater use of inactivated ones due to lack of side effects. The present review discusses the etiology, epidemiology, pathogenesis, and clinical signs of contagious agalactia in small ruminants along with trends and advances in its diagnosis, treatment, vaccination, prevention, and control strategies that will help in countering this disease.

## 1. Introduction

Contagious agalactia, a disease with the involvement of multiple organs, produces systemic infections and is supposed to be among the most serious diseases of small ruminants, produced by mycoplasmas after contagious caprine pleuropneumonia (CCPP) [1–3]. In many parts of the world countries most notably in the Mediterranean basin, are severely affected economically due to outbreaks. It is a listed disease by World Organization for Animal Health (OIE), which is responsible for severe losses to dairy industry [4, 5].* Mycoplasma agalactiae* is the classical etiological agent of this disease which primarily affects goats and sheep along with many wild species. The impressive diffusion of this disease is due to several factors including primitive herding practices, inefficiency of antimicrobial therapies, and adoption of very few prophylactic measures [6]. The disease has been reported from almost all the countries and continents of the world and is responsible for heavy economic losses to shepherds mainly due to high morbidity rather than high mortality in sheep population throughout the world [1, 4, 7–9]. In the European countries major economic losses are incurred upon by the disease due to reduced or suppressed production of milk and abortion along with high morbidity as well as mortality rates in adult sheep. Along with this the cost of diagnosis is a major problem which has been estimated to be approximately 20 million Euros for a year [3, 10].

*Mycoplasma agalactiae* is the second one in mycoplasma species, after* M. mycoides* subsp.* mycoides* type SC. It was first reported, dating back to 1923, when Bridre and Donatien cultivated the microbe responsible for causing contagious agalactia (CA) in goats for the first time [11]. In 1925, Bridre and Donatien for the first time reported CA as a disease of sheep and goats characterized by mastitis, arthritis, and keratoconjunctivitis and succeeded in growing the causal organism [12]. However, the disease was first notified in 1816 in Italy. Initially in 1931, the organism was named as* Anulomyces agalaxie* [13] and after the advent of new taxonomy of mycoplasmas, Freundt named it* Mycoplasma agalactiae* [14]. Initially,* M. agalactiae* was considered to be the classical etiological agent of contagious agalactia [4]. However, now this designation of* M. agalactiae* disease as “contagious agalactia” appears to be misnomer as disease occurs in both sexes. Further the involvement of other species is also well established in mycoplasma induced agalactia. It is because the complex of disease conditions, namely, mastitis, agalactia, keratoconjunctivitis, and pneumonia (MAKePS syndrome), which was earlier assigned to* M. agalactiae* is supposed to be due to the cluster including* M. mycoides* subsp.* mycoides* large colony type (LC),* M. capricolum* subsp.* capricolum*, and* M*.* mycoides* subsp.* capri* [15]. Moreover, a disease with almost similar clinical and pathological manifestations is also caused by* Mycoplasma putrefaciens* in goats [16]. Still* M. agalactiae* is supposed to be the major pathogen which accounts for 90% outbreaks of contagious agalactia syndrome in goats [17] and almost 100% in sheep [18, 19]. Most importantly, control and eradication of contagious agalactia can be obtained through better diagnostic tests and through a more efficient vaccine [20]. The present review discusses some salient features of* M. agalactiae* and the disease (contagious agalactia) caused in small ruminants with regards to epidemiology, pathogenesis, and clinical signs, along with focusing the trends and advances on its diagnosis, treatment, vaccination, prevention, and control strategies that will help in countering this disease in a better way.

## 2. Etiology

### 2.1. Morphology, Cultural and Biochemical Characteristics

*Mycoplasma agalactiae* is a polymorphic bacterium with the size in the range of 124–250 nm and has a very small genome (1 × 10^9^ Da). The isolation of* M. agalactiae* is bit time taking due to slow adaptation of bacterium to new environment. Freshly isolated strains of the bacterium are slow growing, but when adapted to laboratory conditions these grow easily in majority of the commonly used media for mycoplasma growth [21, 22].* M. agalactiae* produces colonies with dark centers producing typical fried-egg appearance and this phenomenon is called as “film and spot”. In biochemical characterization,* M. agalactiae *neither ferments glucose nor hydrolyses urea and arginine [18, 23, 24]. Staining of the mycoplasma colonies is performed with Giemsa stain from solid agar media to observe the colony characteristics [21, 22]. The absence of cell wall in the mycoplasma leads to pink color staining with Gram staining [21, 25].

### 2.2. Growth Requirements

Initially it takes few days to a week time to grow* M. agalactiae* in laboratory media, but the growth time is reduced after adaptation [22, 26]. Moreover, the growth of strains is comparatively slow in solid media in comparison to liquid media [22].* M. agalactiae* is routinely grown at 37°C in laboratory media enriched with sterol [22, 27]. The growth on solid media in humid atmosphere is supported by 5% CO_2_ and the osmotic pressure of 7 to 14 atmospheres [7, 22].* M. agalactiae* multiplies by budding or binary division and grows well on special liquid and solid media with the addition of sterols, which is an essential component for the synthesis of plasma membrane. As the organism is sensitive to alteration in pH, optimum pH should be maintained at 7.6 with the addition of organic components like DNA and NADH to improve the growth [21, 22, 28].

### 2.3. Sensitivity and Resistance

*M. agalactiae* is very sensitive to high temperature and can be easily inactivated with the exposure to 60°C for 5 min. and within a minute at 100°C. It can be inactivated with the direct exposure to sunlight during hot summer season. The survival time of the organisms varies from 1-2 weeks to 3-4 months at room temperature and in refrigerator at 8°C, respectively, depending upon other conditions like pH of media. Humid and cold conditions support its survival. It can survive for 8 to 9 months of period at −20°C. Exposure to ultraviolet radiation and dyes inactivates it quickly. Moreover, the organism can be easily destroyed by commonly used disinfectants such as potassium hydrochloride, formalin, and chloramines [16]. Similar to other mycoplasma species* M. agalactiae* also lacks cell wall and, due to the presence of only the plasma membrane, it is resistant to penicillin and its analogues. However, its cells are sensitive to digitonin. The presence of only plasma membrane makes it vulnerable to osmotic shock and the effect of detergents [21, 29].

### 2.4. Antigenicity

In 1968, Razin [30] applied polyacrylamide gel electrophoresis (PAGE) to study the electrophoretic patterns of mycoplasma cell proteins to resolve several taxonomic problems in the Mycoplasmatales. He observed the similar patterns for several mycoplasmas, namely,* M. mycoides* subsp.* capri*, other caprine mycoplasmas,* M. agalactiae* and* M. agalactiae* var.* bovis*, and different murine mycoplasmas. However, the avian mycoplasma species, namely,* M. gallisepticum*,* M. synoviae*,* M. meleagridis*,* M. gallinarum*, and* M. iners*, showed easily distinguishable and specific patterns.* M. agalactiae* has many cross-reactive antigens of heterogenous nature; hence, initially due to lack of knowledge regarding its protein heterogeneity, it was reported to be a species with uniform antigenicity [31, 32].* M. agalactiae* and* M. bovis* are almost identical in cell and colony form as well as in their metabolic behavior with the sharing of high number of antigens. It is difficult to differentiate them on the basis of usual morphological, metabolical, and serological methods [21, 33–35]. Now the antigenic heterogeneity of* M. agalactiae* has been duly established [22, 36–41]. In a recent study, SDS-PAGE revealed 24 polypeptides in whole cell antigens (WCA) and sonicated supernatant antigen (SSA) of Indian isolates of* M. agalactiae*, respectively. They are in the range of 20.89 to 181.97 kDa with seven major proteins of 63.10, 60.25, 58.88, 47.86, 44.66, 33.88, and 28.84 kDa molecular weights. On immunoblotting with polyclonal rabbit serum produced against* M. agalactiae*, all the major proteins appeared immunogenic with 12 to 14 immunogenic polypeptides [42]. These major immunogenic proteins are being targeted for the development of diagnostic aids for the detection as well as differentiation of* M. agalactiae* from other related mycoplasmas.

## 3. Epidemiology

The disease primarily occurs in Mediterranean countries [43, 44].* M. agalactiae* has been reported to be isolated from different parts of the world in various countries, namely, India [45], Australia [46], Turkey [47], Iran [48], Mongolia [49], Nigeria [50], Senegal [51], Iraq [52], and Spain [3]. Apart from the above it has also been reported from regions and countries such as European litoral, Bulgaria, Serbia, Sudan, Russia, Asia Minor, America, and Switzerland [4, 18, 53]. Thus by the end of the 19th century the disease had become enzootic in many parts of the world [41].

The disease has also been noted in the countries of West Asia; Central as well as North and East Africa; the United States as well as Brazil. In both sheep and goat population of Jordan,* M. agalactiae* is the major pathogen causing the disease contagious agalactia. In the Western Pyrenees basin of France there has been reemergence of this particular pathogen. There is however research gap regarding the epidemiology of the disease in Spain which is among one of the countries of European Union containing large population of sheep [54, 55].

*M. agalactiae* infection represents a risk for population density and maintenance in wild populations, namely,* Iberian ibex* (*Capra pyrenaica*) in Spain [56]. The predisposing factors for the occurrence of the disease are sex (in females), age (young animals), and metapopulation [57]. In the population of wild ibex the strains of* M. agalactiae* have been found to be highly related and appeared to originate from an individual parental clone spreading to another species of wild ungulate (chamois) in same geographical location. Strains found in Europe are clearly different from those found nearby. The pathogenesis of* M. agalactiae* infection is not clear in ibexes, but in Alpine there has been atypical strain emergence. This has given rise to the thought that wild fauna can act potentially as reservoirs of mycoplasmas that are pathogenic [5].

## 4. Transmission of Disease

**Figure 1:**
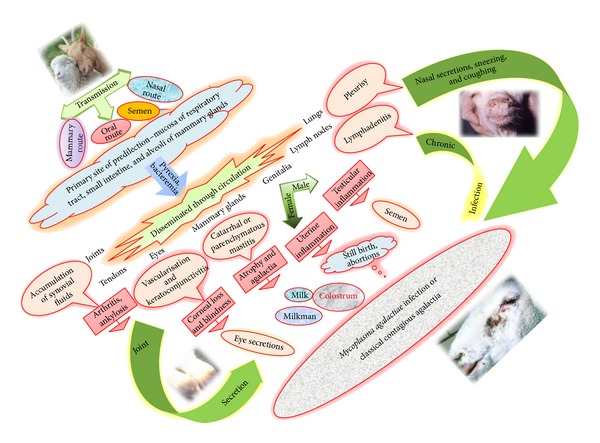
Transmission and pathogenesis of* Mycoplasma agalactiae*.

The sustainability of organism at room temperature supports its rapid spread through contact from infected to healthy animals. The main sources of infection include auricular, ocular and nasal secretions, faeces, milk, urine, and excretions from joint lesions [58]. Sexual transmission through infected male has been reported. Contaminated utensils and milker's hands are vital source of infection. Vertical transmission is observed through contaminated colostrum or milk [18, 59, 60]. The various sources of disease transmission have been depicted in Figure 1.

In majority of cases chronic or persistence infection for several months in flock is observed with clinically positive animals during favorable environmental conditions as at the time of hot and humid summer. Young, malnourished, pregnant, and immunocompromised animals are comparatively more susceptible to the infection [61]. There are reports of excretion of organisms in milk even after 8 years of infection with mild and with or without clinical signs [37, 62]. Thus the presence of asymptomatic carriers in a herd which carry the infectious agent is of major concern. Persistence of antibodies could be observed up to 8 and 3 years of clinical disease in goats and sheep, respectively [63, 64]. Animal species other than homologous hosts as cattle, camel, and many other wild small ruminants can also act as reservoir of the infection. These carrier states are more frequently observed in females, particularly in their genital tracts [16, 56, 65].

## 5. Pathogenesis

*M. agalactiae* is comparatively stable at room temperature and in general is transmitted through oral, respiratory, and mammary route. The different routes of transmission and process of disease development have been depicted in Figure 1. It has been isolated from nasal secretions [21, 66, 67], faecal samples [68], milk [59, 69], and aborted fetus [70]. It suggests that the primary site of predilection is the mucosa of respiratory tract, small intestine, and alveoli of mammary glands, respectively, depending upon the respiratory, oral, and mammary routes [21]. However, as such no disease condition is reported with the involvement of small intestine. Once infection is set up, fever is observed due to bacteremia accompanied by fever. Then following the initial multiplication organisms are disseminated through circulation to different vital organs, namely, lungs, lymph nodes, eyes, mammary glands, joints, and tendons, producing various clinical signs [8, 26]. Involvement of connective tissues in mammary glands leads to initial inflammation which ultimately turns in catarrhal or parenchymatous mastitis leading to atrophy and agalactia [21, 53]. Animals suffering from mastitis can spread disease to young ones through colostrums or milk [71]. In general, lung lesions are observed with* M. agalactiae* infection, although outbreaks of pleurisy among goats with the isolation of mycoplasma have been reported [8, 43]. Painful swelling of joints with the accumulation of synovial fluids leads to arthritis mainly in carpal and tarsal joints. In chronic cases eventual loss of joints leads to ankylosis. Affections in eye cause severe losses of cornea, ultimately leading to blindness through vascularisation and keratoconjunctivitis [26, 60, 72, 73]. Affections of genital organs are also observed with occasional abortions or still births in pregnant animals, mainly due to the inflammation of the uterus. In male animals it may produce testicular inflammation. The association of* M. agalactiae* with granular vulvovaginitis in goats has been observed [8, 74]. Despite all kinds of clinical affection and metabolic alteration,* M. agalactiae* infection in goats does not produce anaemia or septicaemia [26]. However, the presence of mycoplasma in circulation, that is, mycoplasmamia, is mainly responsible for its dissemination in various organs, particularly in sheep. The disease conditions produced by* M. agalactiae* are responsible for high economic losses which are due to the loss of milk and loss of lambs and kids because of abortions, neonatal deaths, and loss of animals themselves. Moreover, losses also occur due to the losses caused by subacute, acute, and chronic forms of disease in the form of physical weaknesses and the clinical complications which affects the animals in infected herd [35, 50, 74, 75].

### 5.1. Pathogenicity in Laboratory Animals

Pathogenicity of* M. agalactiae* in the laboratory is tested experimentally on mice [76]. The young mice are inoculated through intraperitoneal route with 24 hours of grown young cultures. Then after 24, 48, and 96 hours, the tail blood is applied in liquid and solid media. To judge the presence of mycoplasmaemia in liquid and on solid media, color change due to pH alteration in liquid media and presence of fried-egg colonies are observed on solid media [8, 76–78].

## 6. Clinical Signs

*M. agalactiae* can affect both sheep and goats of either sex. The incubation period of the organism varies from few days to few weeks and even up to two months depending upon the route of entry, number and virulence of organisms, and immune status of the animal [8]. Young animals which are deprived of maternal antibodies, weak, debilitated, malnourished, and immunocompromised and animals under stress during and after transportation, under physiological stress like pregnancy, and exposed to extreme climatic conditions are frequently affected. Depending upon such conditions* M. agalactiae* can produce acute, subacute, or chronic form of disease. In some animals atypical or asymptomatic forms have also been reported [36, 71, 79, 80]. Common clinical symptoms include fever, anorexia, lethargy, and unwillingness to follow the herd, followed by the clinical symptoms depending upon the involvement of various organs such as mammary glands, lungs, genitalia, joints, and conjunctiva. Rare abortions in pregnant animals have also been reported [21, 26, 81]. Importantly, fever is common in acute cases and may be accompanied by nervous signs, but both signs are rare in the more frequently observed subacute and chronic infections.* M. agalactiae* may occasionally be found in lung lesions [82]. However occurrence of pneumonia is not a consistent finding. Loss of milk production, discoloration, saltiness, and change of consistency of milk and ultimately agalactia are commonly observed. Young ones receiving infected colostrums and milk might lead to septicaemia, arthritis, or pneumonia with high mortality of the kids [8, 71]. Chronic involvement of joints and severe losses to cornea lead to lameness along with inability to walk or stand and blindness, respectively [72, 73]. The conditions like pleurisy, arthritis, pneumonia, keratoconjunctivitis, and mastitis usually result from infection with* M. mycoides* too because this organismhas one of the widest geographical distributions and is found wherever contagious agalactia is reported [69]. Congenital polyarthritis has also been reported from goat kid [83, 84]. The clinical conditions produced during infection have been elicited in Figure 1.

## 7. Diagnosis

### 7.1. Conventional Diagnosis

**Figure 2:**
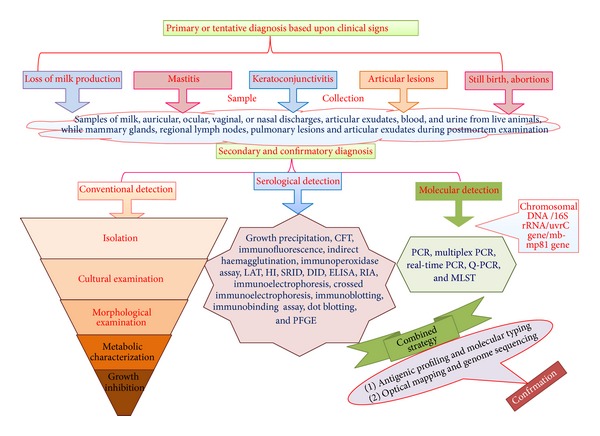
Diagnosis of* Mycoplasma agalactiae* infection.

Primary or tentative diagnosis of the organism is based upon clinical signs, namely, loss of milk production, mastitis, keratoconjunctivitis, and articular lesions. Discoloration of milk in yellowish-green color, ocular discharges, articular swellings, and lameness are suggestive of* M. agalactiae* infection. The clinical diagnosis is confirmed by isolation and identification of the organism in the laboratory [85]. Samples of milk, auricular, ocular, vaginal, or nasal discharges, articular exudates, blood, and urine are used for the diagnosis [21, 26, 86]. For the isolation purposes from infected tissues, samples are collected aseptically from the mammary glands, regional lymph nodes, pulmonary lesions, and articular exudates during postmortem examination [26]. Isolation of* M. agalactiae* from liver, kidney, and spleen could be performed during the phase of mycoplasma. Cultivation is carried out in liquid or on solid media which support mycoplasma growth [18, 21].* M. agalactiae* produces fried-egg colonies. Characterization of isolates based on biochemical tests is not usually recommended [22, 87] due to morphology, growth, and metabolic similarity to some other mycoplasmas [28, 88]. Various methods of diagnosis have been depicted in Figure 2.

### 7.2. Serological Diagnosis

Serological tests of importance for detecting* M. agalactiae* include growth precipitation (GP), immunofluorescence (IF), complement fixation test (CFT), indirect haemagglutination (IHA), haemagglutination inhibition (HI), agglutination, latex agglutination test (LAT), double immunodiffusion (DID), single radial immunodiffusion (SRID), enzyme linked immunosorbent assay (ELISA), radio immunoassay (RIA), and immunoperoxidase (IP) [1, 9, 22, 87, 89–96]. They also include many electrophoretic techniques such as gel electrophoresis, immunoelectrophoresis (IEP), countercurrent immunoelectrophoresis (CCE), and crossed immunoelectrophoresis [22, 40, 97, 98]. Immunoblotting has been used to demonstrate the antigenic specificity by the use of hyperimmune sera from rabbit which is monospecific [42]. Other than these methods, the techniques to separate protein antigens, namely, polyacrylamide gel electrophoresis (PAGE), sodium dodecyl sulphate polyacrylamide gel electrophoresis (SDS-PAGE), two-dimensional immunoelectrophoresis, western blotting, dot blotting, and immunobinding assay, have also been developed and attempted to diagnose caprine agalactia [34, 35, 40, 99–103]. However, to overcome the difficulties and limitations in identification of the organism, diagnosis of* M. agalactiae* can be carried out by the complement fixation test (CFT) or monoclonal antibody based ELISA techniques against individual mycoplasma species or by means of gene amplification techniques [36, 79, 80, 104]. Serological tests have been efficiently used for the diagnosis of contagious agalactia due to* M. agalactiae* from the field cases, but dependency of these tests on crude antigens, in general, may not render them very specific and sensitive. Therefore, many of these tests cannot differentiate between the mycoplasma species due to the presence of common antigens [22, 34, 85, 87]. For differentiation of* M. agalactiae* and* M. mycoides* (large colony) and many other related species monoclonal antibody as well as recombinant protein based ELISA has been described [36, 79, 80, 104]. In small ruminants, affected with contagious agalactia, correlation study conducted on ELISA activity with various other serological tests under the field conditions indicated the ability of the test to detect the subclinical infection caused by the organism and also the ability to screen the goat herds for the presence of carrier animals [9, 61]. The ELISA, CFT, and immunoblotting are supposed to be standard serological tests as per the guidelines of OIE.

### 7.3. Molecular Diagnosis

The recent advances in molecular biology and biotechnology have strengthened the diagnosis, characterization, and differentiation of mycoplasmas including* M. agalactiae*. Cross-reactive closely related species can be certainly differentiated by the use of gene probes, complementary to segments of chromosomal DNA or 16S ribosomal RNA (rRNA) [63, 105, 106] with mixed success (Figure 2). However, the use of polymerase chain reaction (PCR) technique that seems to be even more sensitive and effective tool for the identification purposes is commonly practiced [107]. A simple method for detection of* M. agalactiae* from sheep milk by DNA extraction and subsequent PCR has proven to be faster than cultural isolation of the organism and has reduced the time required for diagnosis from days to hours [39, 108–110]. Use of pulsed-field gel electrophoresis (PFGE) has also strengthened the* M. agalactiae* diagnostics [39, 111]. PCR techniques based on 16S rRNA [79, 112], uvrC gene [113], and multiplex PCR [15, 26, 100, 114, 115] are being routinely used for the identification of* M. agalactiae* and have high diagnostic value (Figure 2). Molecular detection based on uvrC gene is of prime importance according to the recommendation of OIE.

By amplifying the 16S rRNA gene it is possible to identify the* M. agalactiae* isolates by means of PCR and it has been found that 99.8 percent similarity is shared by* M. agalactiae* as well as* M. bovis* isolates. Certain other diagnostic strategies include unknown sequence amplification or amplification of certain particular gene apart from uvrC like mb-mp81 gene encoding the P81 membrane protein. PCR-restriction fragment length polymorphism (PCR-RFLP) technique forms the basis of this method [107, 116]. Real-time quantitative PCR (Q-PCR) assay has been used for quantifying the organism absolutely and is becoming increasingly popular for the purpose of diagnosis in both clinical and food microbiology [117]. Higher specificity as well as sensitivity of analysis is provided by this technique thus reducing the chances of cross-contamination. Chemistry of molecular beacon has been used by certain workers for developing a real-time PCR detection methodology. This method targets a region of 117 base pairs (bp) of the mb-mp81 gene of* M. agalactiae* encoding P81 lipoprotein gene [118]. In each of the reaction mixtures it is mandatory to add internal amplification control (IAC) for assessing the potential inhibitory effect of PCR or thermocycler malfunctioning. It is known that a chimeric nontarget DNA fragment is IAC present in each of the reaction mixtures and target sequence can be used to coamplify it [119]. However, in an another method, alternate template instead of being used in same PCR reaction mixture, it is run under similar amplification conditions in separate PCR wells [118, 120].

Multilocus sequence typing (MLST), a robust molecular tool, has also been used for comparison of genetic sequences of* M. agalactiae* [121, 122].* M. agalactiae* possesses a capacity for phenotypic diversification of its surface antigens [36, 79]. In this regard, analysis of the antigenic variation of several* M. agalactiae* wild strains using different sera from naturally infected sheep followed by characterization of two strongly immunogenic membrane surface proteins of 55 kDa and 35 kDa, respectively [110] is quiet noteworthy. The gene encoding the P48 major surface lipoprotein has been characterized and reported to play a crucial role in the immune response of infected animals. Analysis of a recombinant P48 expressed in* E. coli* by using western blot and indirect ELISA proves to be a diagnostically relevant marker of* M. agalactiae* infection [123].

A combined strategy including antigenic profiling, molecular typing, and optical mapping as well as sequencing of the whole genome has shown the presence of 35 coding sequence. These sequences are based on gene involved, expression of antigens, and vice versa. They are contained in a large prophage and have confirmed the characterization of isolates in wild ungulates [5].

## 8. Treatment

Initial therapy for the infection included the use of arsenicals, particularly sodium and zinc salts of acetarsol. The use of these compounds and their continuous therapeutic usage had adverse effects. Presently all over the world preferred therapy is the use of antibiotics based on drug sensitivity. Commonly used antibiotics include tetracycline, macrolide, clindamycin, florfenicol, tylosin, tiamulin, tilmicosin, and fluoroquinolones [35, 124–126]. Systemic use of antibiotic responds well; however, local application in advanced stages to avoid damages in mammary glands, conjunctiva, and joints should accompany systemic treatment [127, 128]. Fluoroquinolones, particularly enrofloxacin which is converted to ciprofloxacin after metabolism [129], might have less chances of resistance development. Moreover, the peak value of minimum inhibitory concentration (MIC) of ciprofloxacin is reached within few minutes in sheep [129–131] so it would be more useful in acute cases. The use of traditional antibiotics in acute cases is followed by the long acting preparations which have vital role in the subclinical and chronically affected animals. The confirmation and culling policy has limited the use of antibiotics; however precious animals and suspected animals are always treated with parental therapy followed by long acting oily preparations.

## 9. Prevention and Control

The multiple sources of infection and excretion of* Mycoplasma agalactiae* through various body secretions lead to rapid spread of infection. Thus the timely and quick response to the infection is essential for the prevention and control of the spread of infection to susceptible animals [35].* M. agalactiae* infection could be prevented by adopting good managemental practices and following continuous surveillance/monitoring for the pathogen. Many times subclinically infected animals may also spread the infection; hence there is always a need to apply specific, sensitive and rapid diagnostic procedure for its early detection. Till confirmation suspected animals should be isolated and kept under observation. Immediately after disease confirmation, culling of all the contact and affected animals is recommended. Proper disposal of litter and other materials, namely, discharges and aborted fetus, and proper sterilization of contaminated utensils are recommended. Use of disinfectants as hypochloric acid, formalin, cresols, and phenolic substances along with commonly used quaternary ammonium compounds is effective against the organism [21, 35]. Proper screening of the semen for artificial insemination and bucks to be used should be conducted on regular basis. To avoid the vertical transmission ewes should be vaccinated. In endemic areas vaccination with locally developed vaccine is effectively applied throughout world.

## 10. Vaccines

Similar to many other bacterial agents, both live attenuated and inactivated vaccines are available for caprine agalactia [2, 111, 132–140]. These vaccines are both safe and effective [68, 137, 138, 141, 142]. The vaccination against* Mycoplasma agalactiae* in sheep induces both specific and nonspecific, humoral, and cellular response irrespective of type of vaccine. However, duration of persistence of antibodies depends upon multiple factors, namely, strain used, adjuvant incorporated, dose of vaccine, routes of inoculation, physiological status of animal, and so forth. Live attenuated vaccines are more effective and have been reported to provide better protection in ewes and their lambs than the inactivated vaccines but can produce a transient infection with shedding of mycoplasma through milk. Importantly, the live vaccines should be part of a regional plan in which all flocks from which animals are likely to come into contact be vaccinated at the same time. Inactivated vaccines are much safer with no side effects but have shorter period of protection with doubted efficacy [71, 140, 143]. It is possible that in some instances the apparent lack of protection given by vaccines can be the result of infection of animals with one of the other four mycoplasmas involved in the contagious agalactia syndrome. The issue for vaccine is duration, the levels of immunity are being addressed, and a combined inactivated vaccine with aluminium hydroxide gel and saponin and with mineral oil as adjuvant was also attempted in laboratory [139, 140] and field condition [137, 138, 144]. A saponified vaccine was reported to be effective in initial laboratory trial in mice [145]. Three inactivated vaccines, namely, A, B, and C, with several adjuvants (oil-emulsified) prepared with* M. agalactiae* have been evaluated for immunogenicity as well as efficacy purposes. For this purpose, animals have been divided into three groups and immunized with same vaccine using different adjuvant. After challenge with the organism, clinical protection has been induced by all the vaccine formulations. Full protection however has been induced by only the vaccine C which contains Montanide ISA-563 as well as Marcol-52 and Montane-80 (ratio: 30% : 63% : 7%) and has been found to induce protection at full level in animals that are challenged. This has helped in preventing both the clinical signs' onset and infection [136]. Out of available live attenuated and adjuvant (alum precipitated or saponified) vaccines, inactivated vaccines are of greater use due to lack of side effects. However, the protection period is short in comparison to live vaccines.

## 11. Conclusion and Future Perspectives

Contagious agalactia is considered as a neglected disease of small ruminants because of the complex disease distribution pattern, ubiquitous nature of the causal agent, and poor sheep and goat farm managemental practices, especially in developing and underdeveloped countries like India. Rapid spread and multiple sources of infection along with vertical and horizontal mode of transmission are matter of immense concern and severely affect the local economy. Depending upon conditions like deprivation of maternal antibodies, immunocompromised state, stress due to transportation, pregnancy, or extreme climatic conditions, animals may suffer from acute, subacute, chronic, or asymptomatic forms of disease. The isolation of* M. agalactiae* is a difficult task due to its property of lack of cell wall resembling other organisms of the genus* Mycoplasma*, and serological tests can efficiently identify* M. agalactiae*. However, in recent years, the isolation of* M. capricolum* subsp.* capricolum* (*Mcc*) and* M. mycoides *subsp.* capri* (formerly* M. mycoides* subsp.* mycoides*; large colonies) from sheep and goat having mastitis and arthritis complicated the situation. The cross-reactivity of many antigens of these mycoplasmas may lead to false reactivity. Under such conditions, the advanced molecular detection techniques like 16S rRNA based PCR and multiplex PCR certainly help in differentiation of closely related species of the organism which often cause confusion in the mind of the diagnostician. Systemic uses of tetracycline, macrolide, or quinolone group of antibiotics along with local application in advanced stages are useful treatment options. Good managemental practices like isolation of sick animals along with test and slaughter policy form the basis of disease prevention. For the prevention and control of disease in particular to endemic areas vaccination is only effective strategy. Mono-, bi-, and trivalent live attenuated and adjuvant inactivated vaccines are available with local strains with limited success rate. However, further research on the molecular epidemiology of the organism in both domestic and wild animals is necessary to fully understand the disease distribution pattern to effectively manage the populations of goat and sheep and protect them against the infection. Similarly, there is need to explore the advancements made in the field of vaccinology for the management of the disease more efficiently in sheep and goat population.
